# Natural Chalcones and Derivatives in Colon Cancer: Pre-Clinical Challenges and the Promise of Chalcone-Based Nanoparticles

**DOI:** 10.3390/pharmaceutics15122718

**Published:** 2023-12-01

**Authors:** Soufyane Hba, Suzan Ghaddar, Hicham Wahnou, Aline Pinon, Riad El Kebbaj, Christelle Pouget, Vincent Sol, Bertrand Liagre, Mounia Oudghiri, Youness Limami

**Affiliations:** 1 Laboratory of Immunology and Biodiversity, Faculty of Sciences Ain Chock, Hassan II University, B.P 2693 Maarif, Casablanca 20100, Morocco; hbasoufyane@gmail.com (S.H.); hwwahnou@gmail.com (H.W.); mounia.oudghiri@univh2c.ma (M.O.); 2 Univ. Limoges, LABCiS, UR 22722, F-87000 Limoges, France; suzan.ghaddar@etu.unilim.fr (S.G.); aline.pinon@unilim.fr (A.P.); christelle.pouget@unilim.fr (C.P.); vincent.sol@unilim.fr (V.S.); 3 Laboratory of Health Sciences and Technologies, Higher Institute of Health Sciences, Hassan First University of Settat, Settat 26000, Morocco; elkebbajriad@gmail.com

**Keywords:** natural chalcones, colon cancer, chalcones-based nanoparticles, drug delivery system

## Abstract

Colon cancer poses a complex and substantial global health challenge, necessitating innovative therapeutic approaches. Chalcones, a versatile class of compounds with diverse pharmacological properties, have emerged as promising candidates for addressing colon cancer. Their ability to modulate pivotal signaling pathways in the development and progression of colon cancer makes them invaluable as targeted therapeutics. Nevertheless, it is crucial to recognize that although chalcones exhibit promise, further pre-clinical studies are required to validate their efficacy and safety. The journey toward effective colon cancer treatment is multifaceted, involving considerations such as optimizing the sequencing of therapeutic agents, comprehending the resistance mechanisms, and exploring combination therapies incorporating chalcones. Furthermore, the integration of nanoparticle-based drug delivery systems presents a novel avenue for enhancing the effectiveness of chalcones in colon cancer treatment. This review delves into the mechanisms of action of natural chalcones and some derivatives. It highlights the challenges associated with their use in pre-clinical studies, while also underscoring the advantages of employing chalcone-based nanoparticles for the treatment of colon cancer.

## 1. Introduction

In 2023, a concerning statistical forecast indicated that approximately 153,020 individuals will receive a diagnosis of colorectal cancer (CRC), while 52,550 lives will tragically be claimed by this disease [1]. Notably, this includes 19,550 cases and 3750 deaths among individuals younger than 50, underscoring this healthcare challenge’s pressing significance [1]. Colon cancer, also known as CRC, affects the colon or rectum, which are both critical digestive system components [2]. CRC arises when abnormal cells in the colon or rectum divide uncontrollably, forming malignant tumors. Colon cancer is one of the most common cancers worldwide, and its incidence continues to rise [2]. It imposes a considerable burden on healthcare systems and patients alike, necessitating the development of innovative and effective therapeutic strategies. Given the diverse mechanisms and pathways involved in its tumorigenesis, colon cancer demands a multifaceted approach to treatment.

**Figure 1:**
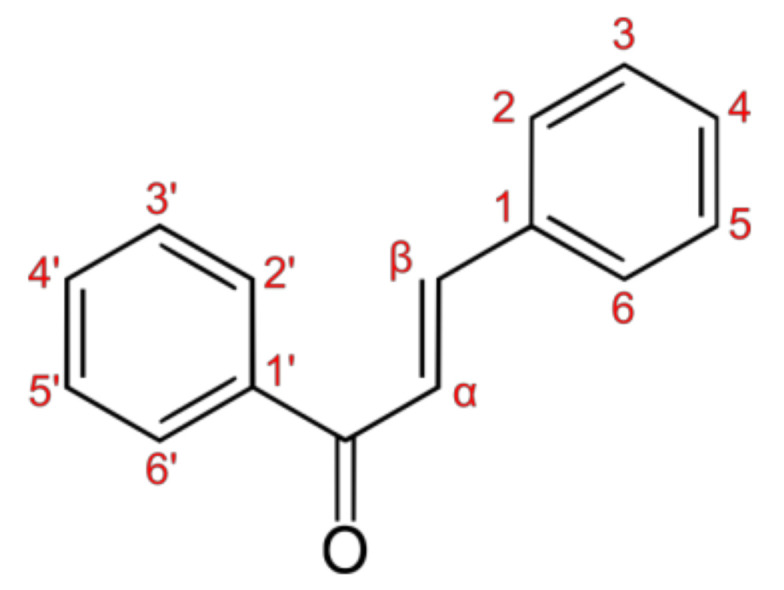
General skeleton of the chemical structure of chalcones.

In recent years, one promising avenue in colon cancer treatment has emerged in the form of chalcones, a class of compounds with diverse pharmacological activities. Chalcones have garnered attention for their potential to modulate the multiple signaling pathways implicated in CRC development and progression [3]. Chalcones (except for curcumin, which may be considered a bis-chalcone derivative) are characterized by their chemical structure, which consists of two aromatic rings linked by a three-carbon α,β-unsaturated carbonyl system [4] (Figure 1). This unique structure allows them to interact with specific molecular targets within the cancer cells. They have demonstrated significant anticancer properties, making them attractive candidates for targeted therapeutics in the fight against colon cancer [3].

This review will focus on utilizing natural chalcones and some derivatives (such as curcumin) as targeted therapeutics for colon cancer, exploring their intriguing anticancer properties and mechanisms of action. Then, it will shed light on several critical aspects of CRC treatment by examining its poor solubility, identifying optimal sequencing strategies for administering therapeutic agents, delving into the resistance mechanisms that challenge treatment efficacy, and exploring the potential of combination therapies involving chalcones. Moreover, it will delve into the integration of nanoparticle-based drug delivery systems to enhance the efficacy of chalcones.

Through a deeper understanding of these approaches, we aim to shed light on the potential breakthroughs in colon cancer treatment and their broader implications for oncology. By harnessing the therapeutic potential of chalcones and integrating advanced drug delivery systems, we hope to pave the way for more effective and personalized treatments for colon cancer patients, ultimately improving their prognosis and quality of life.

## 2. Anticancer Activity of Chalcones against Colon Cancer

**Figure 2:**
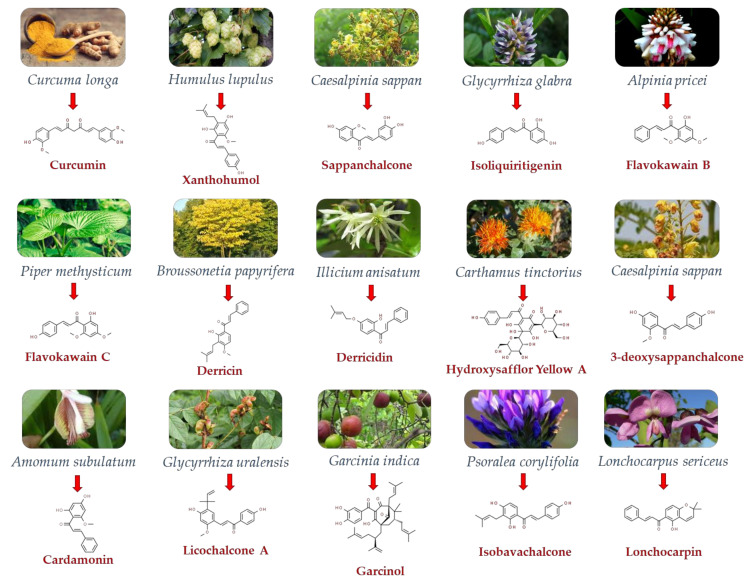
Plant origin of some natural chalcones and derivatives, with their 3D chemical structures (obtained from pubchem.ncbi.nlm.nih.gov (accessed on 20 October 2023)).

This section delves into the exciting realm of natural chalcones and some derivatives, highlighting their potential anticancer activity. Figure 2 and Table 1 specifically focus on their role in addressing colon cancer and describing the researchers’ findings.

### 2.1. Curcumin

**Figure 3:**
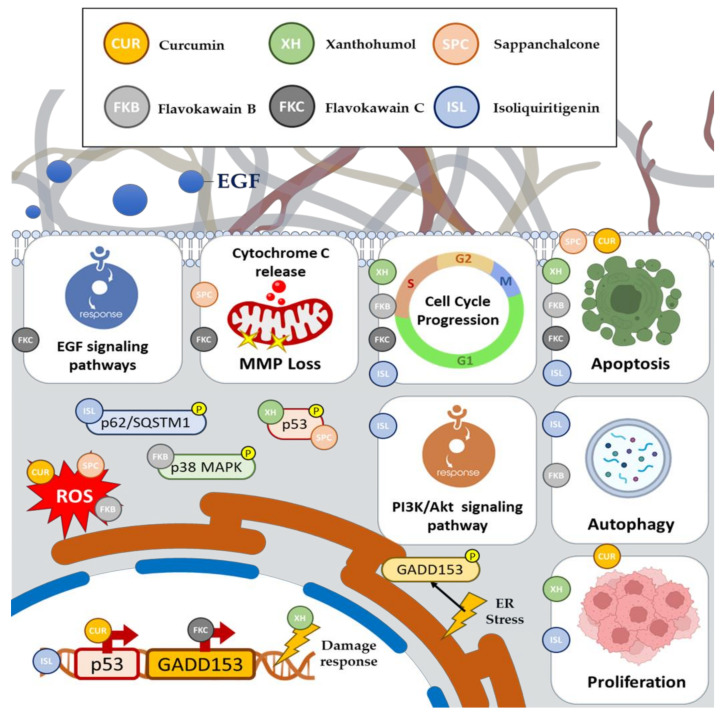
Anticancer mechanisms of action of curcumin, xanthohumol, sappanchalcone flavokawain B, flavokawain C, and isoliquiritigenin.

Curcumin (CUR), which may be considered a bis-chalcone derivative [5], is a natural compound found in *Curcuma longa*, a flowering plant native to South Asia that is known for its rhizomes, which are ground to produce the spice known as turmeric [6]. CUR is a mixture of 3 compounds, the main structure of which is (1*E*,6*E*)-1,7-bis (4-hydroxy-3-methoxyphenyl) -1,6- heptadiene-3,5-dione (or curcumin I). CUR, which is responsible for turmeric’s color and health benefits, has potential therapeutic properties [7] (Figure 3).

Shehzad et al. revealed that CUR effectively inhibited the proliferation of HCT-15 cells and induced apoptosis in a dose- and time-dependent manner. The morphological and biochemical features of apoptosis and the generation of reactive oxygen species (ROS) were observed in cells treated with 30 and 50 μM of CUR [8]. Furthermore, CUR treatment activated caspase-3 and downregulated p53 mRNA expression and pre-mRNA processing factor 4B (Prp4B) in a time-dependent manner [7,8]. The transfection of HCT-15 cells with a Prp4B clone disrupted the growth inhibition caused by 30 μM of CUR. Subsequent cell fractionation revealed the translocation of Prp4B from the cytoplasm to the nucleus. The knockdown of Prp4B with siRNA diminished the protective effects of Prp4B against CUR-induced apoptosis [8].

### 2.2. Xanthohumol

Xanthohumol (XH) is a naturally occurring prenylated chalcone that is frequently extracted from the hop plant (*Humulus lupulus*) and is renowned for its multifaceted array of biological effects [9], encompassing various biological effects such as antiviral, antimicrobial, anti-inflammatory, and immunomodulatory functions [10] (Figure 3).

Previous research has examined the impact of XH on CRC inhibition or eradication. In a study by Liu et al., XH demonstrated a significant anti-tumor effect on CRC by reducing HK2 expression and glycolysis. XH effectively inhibited CRC cell growth in both in vitro and in vivo models [11]. Additionally, XH treatment stimulated cytochrome C release and activated the intrinsic apoptosis pathway [11,12]. Furthermore, the study findings indicated that XH downregulated the EGFR-Akt signaling pathway. When constitutively activated Akt1 was overexpressed exogenously, it notably compromised XH-induced glycolysis suppression and apoptosis induction [11].

A separate investigation has provided initial evidence indicating that XH activates the DNA damage response (DDR) and triggers apoptosis [13]. XH demonstrates the potential to sensitize CRC cells such as SW480 that are resistant to conventional anticancer drugs such as SN38, which is known for its role in chemotherapy [13,14]. XH activates the key pathways involving the ATM and ATR proteins, which play crucial roles in DDR [15,16]. Interestingly, XH‘s impact on the cell cycle varies among the different CRC cell lines. In SW620 and HT-29 cells, XH disrupts the cell cycle’s G0/G1 and S phases, affecting the cyclins and cyclin-dependent kinases (CDKs) [13]. Conversely, in metastatic colorectal SW620 cells, XH primarily increases the DNA replication phases. In the most resistant SW480 cells, XH induces significant apoptosis. Moreover, XH activates p53 and p21, key factors in the DNA damage response, which can enhance the effectiveness of chemotherapy drugs such as SN38 [17,18]. The study suggests that XH pretreatment may improve the efficacy of chemotherapy and reduce its toxicity.

### 2.3. Sappanchalcone

Sappanchalcone (SPC) is a natural compound derived from the heartwood of the Sappan tree (*Caesalpinia sappan*), which is native to Southeast Asia [19]. This phytochemical has emerged as a subject of interest in cancer research due to its cytotoxic effects on various cancer cell lines, including colon cancer (Figure 3).

Studies have explored the cytotoxic effects of SPC on colon cancer cells, particularly HCT116 and SW480 cells with different p53 statuses [20]. The study demonstrates that SPC inhibits the growth of both cell lines, with HCT116 cells being more sensitive [20]. It induces apoptosis in both cell lines via the caspase-dependent and caspase-independent pathways [20]. SPC disrupts the mitochondrial membrane potential, regulates Bcl-2 family proteins, and increases ROS production, leading to apoptosis. In HCT116 cells, SPC activates p53, suggesting a p53-associated apoptotic mechanism, whereas this effect is absent in SW480 cells, due to the lack of significant changes in cleaved caspase expression [20].

### 2.4. Isoliquiritigenin

Isoliquiritigenin (ISL) is a natural compound with a simple chalcone structure that belongs to the flavonoid group. It is known for its various potential health benefits and is found in a variety of plant sources, primarily in the roots of licorice (*Glycyrrhiza glabra*) and some other plants [21,22]. Various in vitro studies have explored its anticancer activity, suggesting that ISL may have the potential to inhibit the growth of cancer cells and induce apoptosis, making it a subject of interest in cancer research [23].

In fact, ISL was found to induce G2 cell cycle arrest [24], and to have an effect on death-associated protein kinase 1 (DAPK1) promoter methylation in the colon cancer cell line, indicating its role in influencing the epigenetic regulation of genes associated with cancer [22]. Further in vitro studies revealed that ISL-mediated p62/sequestosome 1 (SQSTM1) induction regulated the apoptotic potential by attenuating caspase-8 activation in CRC cells, providing insights into its mechanisms of action [25], Huang et al. also reported that ISL inhibits the growth of the CRC cell line by suppressing the PI3K/Akt pathway, which is a well-established pathway known for its role in promoting cancer cell proliferation [26].

### 2.5. Flavokawains

Flavokawains B and C, the most studied chalcone compounds in the context of CRC treatment, are intriguing substances with significant potential in the field of cancer research. They are both naturally occurring chalcone compounds, each derived from different plant sources and showing unique mechanisms of action in combating cancer.

#### 2.5.1. Flavokawain B

Flavokawain B (FKB) is a naturally occurring compound derived from the roots of *Alpinia pricei*, a plant native to specific regions, including Taiwan [27,28]. This chalcone compound is part of the flavonoid family and is known for its bioactive properties and potential as an anticancer agent [28]. It is one of the constituents found in the extracts from the rhizomes of this plant, which has gained attention for its medicinal properties, particularly its ability to inhibit the growth of cancer cells and induce various cellular processes related to cancer treatment [27,28,29] (Figure 3).

Researchers revealed that FKB effectively inhibited the growth of HCT116 colon cancer cells by inducing G2/M cell cycle arrest, autophagy, and apoptosis [27]. Notably, it was found that FKB triggered apoptosis by increasing intracellular ROS levels, activating p38 MAPK, and upregulating GADD153 expression [27]. GADD153, in turn, influenced the levels of the Bcl-2 family of proteins, leading to potential mitochondrial membrane loss and apoptosis. The study also explored the link between FKB-induced endoplasmic reticulum (ER) stress and the intrinsic apoptotic pathway. Moreover, FKB was observed to induce ROS generation, which is essential for GADD153 upregulation and apoptosis, while also inducing autophagy [27].

#### 2.5.2. Flavokawain C

Flavokawain C (FKC) is a bioactive compound with a fascinating origin that is deeply rooted in nature. This natural compound is primarily found in the kava plant (*Piper methysticum*), which is native to the South Pacific region. Kava has a long history of traditional use in this region for its calming and stress-reducing effects when consumed as a beverage [30] (Figure 3).

Recent studies have explored its interesting anticancer activity. Researchers investigated the growth-inhibitory and apoptosis-inducing effects of FKC on human cancer cell lines, particularly HCT116 carcinoma cells, while it showed minimal cytotoxicity toward normal colon cells [31]. The study also examined a structurally related compound, gymnogrammene (GMM), for comparison, revealing that FKC exerted pronounced cytotoxicity against HCT116 cells, while GMM had no such effect. This underscored the importance of structural variations in these compounds and their cytotoxicity. The molecular mechanisms of FKC-induced apoptosis were explored, involving the intrinsic and extrinsic pathways. FKC influenced the intrinsic pathway by modifying the expression of Bcl-2 family proteins, Bak and Bax, resulting in mitochondrial membrane permeabilization and the release of apoptogenic proteins such as cytochrome C, Smac/DIABLO, and apoptosis-inducing factor (AIF) [31]. Extrinsic pathway activation was mediated by FKC through increased death receptor levels (DR4 and DR5) and the downregulation of c-FLIP_L_, along with the activation of caspase-8, caspase-9, and caspase-3 [31]. FKC also disrupted the cell cycle by regulating proteins such as CDK2, CDK4, p21Cip, and p27Kip, causing S-phase arrest. Additionally, FKC-induced ER stress was evident from the elevated CHOP levels [31]. The study examined the PI3K/Akt and MAPK pathways, noting that FKC inhibited Akt while promoting ERK 1/2 phosphorylation [31]. Furthermore, another study showed that FKC downregulates numerous inhibitors of apoptosis (IAPs) such as XIAP, c-IAP1, and c-IAP2, increasing cancer cell sensitivity to apoptosis [32]. Additionally, the study suggests that FKC may initiate apoptosis through ER stress since it induces the expression of the GADD153 gene. FKC also increased ROS production and inhibited superoxide dismutase (SOD) activity, potentially contributing to apoptosis by damaging the mitochondrial membranes and activating caspases [32].

The effect of FKC on p53, p21, and p27 was also explored. While FKC upregulated p21 and p27, it had varying effects on p53, depending on the cell type. In HCT116 cells with wild-type p53, the p53 levels initially increased but decreased after 12 h of treatment. In HT-29 cells with a mutated p53, FKC reduced the p53 levels. The reduction of mutant p53 in HT-29 cells may contribute to growth arrest, although the mechanism remains unclear [32].

### 2.6. Derricin and Derricidin

**Figure 4:**
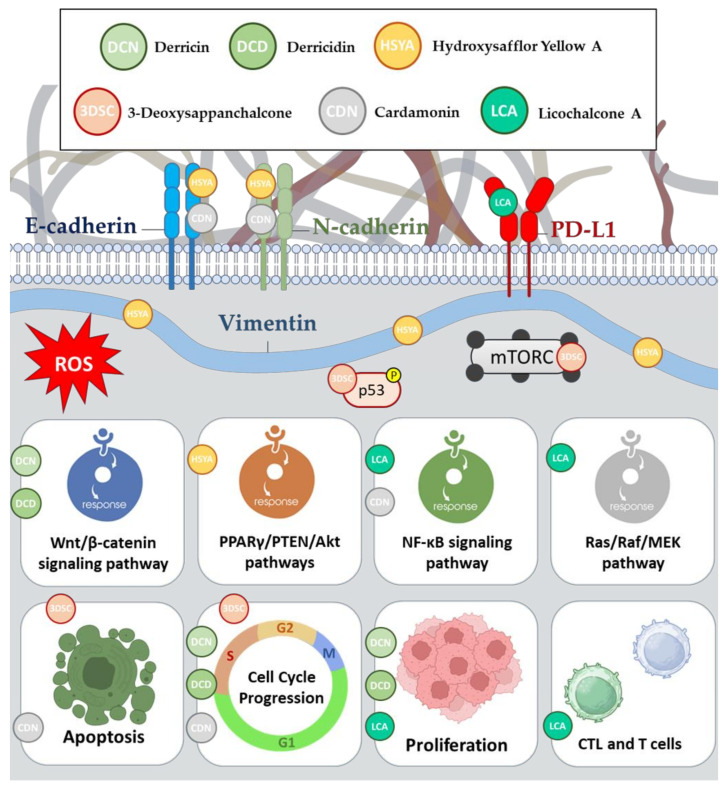
Anticancer mechanisms of action of derricin, derricidin, hydroxysafflor yellow A, 3-deoxysappanchalcone, cardamonin, and licochalcone A.

Derricin (DCN) and derricidin (DCD) are flavonoids belonging to the chalcone subclass [33]. These compounds are natural plant-derived chemicals with similar chemical structures. They have been studied for their potential therapeutic properties, particularly in the context of cancer research (Figure 4).

A previous study demonstrated that both DCN and DCD exhibited strong anti-proliferative effects on the CRC cell lines, HCT116 and DLD-1, by inhibiting cell growth and affecting the cell cycle [33]. Importantly, these effects were primarily observed in CRC cells, suggesting selectivity. The study also explored the potential mechanism behind these effects and found that the flavonoids modulated the Wnt/β-catenin signaling pathway, a pathway that is commonly associated with CRC progression [33]. These findings highlight the potential of DCN and DCD as modulators of the Wnt pathway and raise questions about their interactions with specific components of this pathway and their structure–activity relationships, which require further investigation. Additionally, the study conducted in vivo experiments with *Xenopus* embryos, further supporting the concept of flavonoids’ ability to impact the Wnt/β-catenin pathway [33].

### 2.7. Hydroxysafflor Yellow A

Hydroxysafflor Yellow A (HSYA) is a natural compound found in *Carthamus tinctorius* and has gained attention for its potential therapeutic applications, particularly in cancer treatment (Figure 4).

The anticancer potential of HSYA in CRC was investigated in vitro, focusing on the underlying molecular mechanisms. HSYA demonstrated concentration-dependent inhibitory effects on CRC cell proliferation, migration, and invasion while promoting apoptosis [34]. These actions were associated with regulating the EMT markers, such as the upregulation of E-cadherin and the downregulation of N-cadherin and vimentin. Additionally, HSYA was found to activate the PPARγ/PTEN/Akt signaling pathway, with increased expression of PPARγ and PTEN and decreased phosphorylation of Akt in CRC cells [34]. The role of PPARγ in mediating PTEN expression and subsequently inhibiting the PI3K/Akt pathway was highlighted. The study also revealed that inhibiting PPARγ with GW9662 or the PPARγ knockdown reversed the anticancer effects of HSYA on CRC cells, implicating PPARγ as a key player in HSYA’s therapeutic action [34]. These findings suggest that HSYA holds promise as a potential candidate for CRC therapy by targeting the PPARγ/PTEN/Akt signaling pathway. However, further research is needed to explore its full therapeutic potential and validate these results in additional CRC cell lines and animal models.

### 2.8. The 3-deoxysappanchalcone Compound

The compound 3-DSC, which is short for 3-deoxysappanchalcone, is a natural compound derived from *Caesalpinia sappan* L. [35]. This compound has gained attention due to its potential therapeutic properties, particularly in the context of cancer treatment (Figure 4).

An in vitro study conducted by Zhao et al. focused on the compound’s potential anticancer properties against CRC [36]. The abnormal signaling of T-LAK cell-originated protein kinase (TOPK) is associated with various cancers, including CRC, and has been considered as a therapeutic target [37]. Although previous TOPK inhibitors had several limitations, 3-DSC was identified as a promising candidate [36]. The research demonstrated that 3-DSC specifically inhibits TOPK activity, inhibiting CRC cell growth, cell cycle arrest, and apoptosis. Importantly, 3-DSC showed selectivity for cancer cells, sparing the normal colon cells. This specificity is linked to its ability to induce apoptosis in CRC cells with wild-type p53 while sparing those with mutant p53 [36]. Furthermore, 3-DSC interfered with the downstream signaling pathways and may hold promise as a chemotherapeutic agent for human colon cancers, although further animal studies are needed to confirm its efficacy.

### 2.9. Cardamonin

Cardamonin (CDN), a compound derived from traditional Chinese medicine that is primarily found in the seeds of black cardamom (*Amomum subulatum*), exhibits promising effects in the treatment of chemotherapy-resistant colon cancer [38] (Figure 4).

Studies have shown that CDN significantly reduces cell viability and induces apoptosis in resistant cancer cells, potentially overcoming chemotherapy resistance [39]. Furthermore, CDN suppresses the expression of the key proteins associated with cancer growth and proliferation, including c-Myc and Oct4. Additionally, it inhibits the NF-κB signaling pathway, which is linked to oncogenesis and chemotherapy resistance [39].

Furthermore, researchers investigated the molecular mechanism of CDN in inhibiting lung metastasis in CRC and its potential as a therapy for CRC patients [40]. They found that CDN effectively suppressed ADRB2 expression, leading to reduced viability, migration, invasion, and EMT in CRC cell lines (HT-29 and HCT116). These in vitro findings were validated in a mouse metastasis model, highlighting CDN’s promise as a therapy for lung metastasis in CRC patients [40]. The study also revealed that EMT, a process that is crucial for cancer metastasis, involved the regulation of key markers such as E-cadherin, N-cadherin, MMP-2, and MMP-9, all of which were influenced by CDN treatment. Additionally, the researchers demonstrated that ADRB2 played a role in CDN’s inhibitory effects on CRC metastasis, further suggesting CDN as a molecularly targeted therapeutic drug [40].

### 2.10. Licochalcone A

Licochalcone A (LCA) is a bioactive compound that is naturally found in certain plants, particularly in the roots of licorice plants (*Glycyrrhiza* species). *Glycyrrhiza uralensis* Fisch. ex DC, commonly known as licorice, is a primary source of LCA. This compound has garnered attention for its potential medicinal properties and has been the subject of research in various fields, including its use in cancer therapy and anti-inflammatory applications [41,42,43] (Figure 4).

Researchers evaluated the effects of LCA on specific proteins and pathways [44]. The results, both in vitro and in vivo in a xenograft mouse model, showed the ability of LCA to significantly suppress PD-L1 expression, a vital immune checkpoint molecule often upregulated in various human tumor cells. Moreover, LCA inhibited the NF-κB signaling pathway, which is crucial in cancer cell survival, inflammation, and immunity, and affected the Ras/Raf/MEK pathway [44], which is known for its role in cell growth and cancer [45]. Furthermore, LCA enhanced cytotoxic T lymphocyte (CTL) activity and restored the T cells’ ability to combat tumor cells, potentially boosting the immune response against cancer [44]. Additionally, LCA promoted apoptosis in cancer cells by increasing the expression of cleaved PARP and cleaved caspase-8, which are key proteins involved in programmed cell death [44]. These multifaceted effects underscore the potential of LCA as a therapeutic agent in cancer treatment.

### 2.11. Garcinol

**Figure 5:**
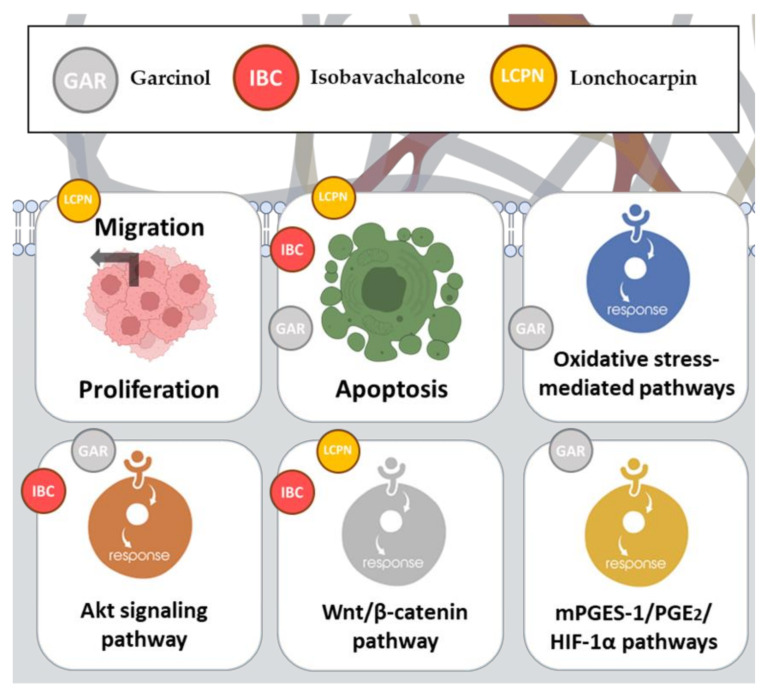
Anticancer mechanisms of action of garcinol, isobavachalcone, and lonchocarpin.

Garcinol (GAR), a chalcone derivative, is a natural compound renowned for its anti-inflammatory and anti-carcinogenic properties. It has been the focus of recent studies investigating its effects on cell growth in colon cancer cells and immortalized intestinal cells [46,47] (Figure 5).

In one study, garcinol and its derivatives, garcim-1 and garcim-2, displayed potent inhibition of colon cancer cell growth. It induced apoptosis in vitro in HT-29 and HCT-116 cell lines, as evidenced by increased caspase activity and annexin V binding [46]. Interestingly, the impact of GAR on cell growth was notably influenced by the presence of serum in the culture medium, with more pronounced inhibitory effects being observed under serum-free conditions. In contrast, GAR promoted cell proliferation at lower concentrations through ROS-mediated pathways, specifically those involving Akt and ERK 1/2 activation [46].

Another study delved into chemoprevention, which has become important as a promising strategy to disrupt cancer initiation and progression [47]. Various compounds, including minerals, vitamins, phytochemicals, and synthetic substances, have been recognized as potential chemopreventive agents [48]. In this context, GAR treatment was explored for its impact on the molecular mechanisms related to apoptosis and migration in HT-29 colon cancer cells. The results demonstrated that GAR treatment significantly reduced the expression of mPGES-1, consequently leading to a decrease in prostaglandin E_2_ (PGE_2_) production, which is a crucial factor in cancer initiation and progression [49]. This inhibition aligned with previous findings highlighting GAR’s ability to inhibit mPGES-1 activity.

Moreover, GAR exhibited the dose-dependent inhibition of HT-29 cell growth over time, along with an induction of apoptosis, as indicated by increased apoptotic cell counts and caspase-3 activation [47]. Notably, GAR also downregulated the expression of key genes involved in angiogenesis, migration, and invasiveness, including HIF-1α, CXCR4, VEGF, and MMP-2/9. These findings collectively suggest that GAR may hold promise as a chemopreventive agent for CRC, primarily through its modulation of the mPGES-1/PGE_2_/HIF-1α pathways [47]. Further exploration of these results in vivo and across different tissue contexts is essential to fully understand the implications of GAR in cancer prevention.

### 2.12. Isobavachalcone

Isobavachalcone (IBC) is a bioactive molecule derived from *Psoralea corylifolia*, a well-known traditional Chinese medicinal herb. IBC has garnered significant attention for its potential anticancer properties and ability to modulate various cellular pathways involved in cancer progression (Figure 5). In a duration- and dose-dependent manner, IBC demonstrated significant cytotoxicity against CRC cell lines, including SW480 and HCT116 [50]. Morphological changes and decreased cell viability were observed in IBC-treated cells, which was consistent with previous findings showing IBC’s inhibitory effects on tumor cell growth [50].

Furthermore, IBC-induced apoptosis was evidenced by its characteristic apoptotic features, such as chromatin condensation, nuclear morphological changes, and Annexin V-FITC/PI staining. Caspase-3 activation and PARP cleavage further supported the induction of apoptosis by IBC. IBC also modulated the Bcl-2 family of proteins with increased Bax expression and translocation to the mitochondria, resulting in apoptosis [50]. Moreover, IBC downregulated the expression of two IAPs, XIAP and survivin, contributing to apoptosis induction through the mitochondrial pathway.

Additionally, IBC hindered the Wnt/β-catenin pathway, a crucial signaling pathway in CRC carcinogenesis, by decreasing the levels of total β-catenin and inducing its phosphorylation. This action was linked to the inhibition of Akt/GSK-3β signaling, highlighting the potential of IBC as a promising therapeutic agent for CRC by targeting multiple cancer-related pathways [50].

### 2.13. Lonchocarpin

Lonchocarpin (LCPN), a chalcone compound, is derived from the *Lonchocarpus sericeus* plant, often referred to as the “Lancepod” or “Yopo” tree, which is native to various regions in Central and South America [51]. LCPN has displayed significant potential in several research studies, particularly regarding its role as a negative modulator of the Wnt/β-catenin pathway and its prospects as an anticancer agent [52].

A study conducted by Predes et al. demonstrated that LCPN acts as a negative modulator of the Wnt/β-catenin pathway in colon tumor cell lines. Notably, the study revealed that LCPN’s influence occurs downstream of β-catenin stabilization, likely at the TCF level [52]. Furthermore, LCPN has demonstrated an impact on cell proliferation, migration, and viability in CRC cell lines (HCT116, SW480, and DLD-1), while exhibiting no effect on a non-tumoral intestinal cell line (IEC-6) [52]. Additionally, in an inflammation-associated CRC mouse model (AOM/DSS), the administration of lonchocarpin resulted in a reduction in cell proliferation within fully developed carcinomas [52]. While these findings are promising, further optimization or alternative administration approaches may further enhance the efficacy of LCPN.

**Table 1:** Mechanisms of the anti-proliferative action of natural chalcones and derivatives against colon cancer.

Chalcone	Model	Mechanisms	References
Xanthohumol	FHC, CCD841 CoN, HT-29, SW480, LOVO, HCT116, and SW620 cell lines Human colorectal tissue BC05118e	Decreased phosphorylation of Akt, expression of Ki67, and HK2; Downregulation of aerobic glycolysis; Induction of the intrinsic apoptosis pathway; Reduced tumor volume and weight; Activation of the DNA damage response; Inhibition of cyclins and CDKs; Activation of p53 and p21.	[11,13]
	Xenograft mouse model *		
Sappanchalcone	HCT116, SW480 cell lines	Induction of caspase-dependent and caspase-independent apoptosis pathways; Disruption of MMP; Regulation of Bcl-2 family; Increase in ROS production; Activation of phospho-p53 and p53.	[20]
Isoliquiritigenin	HCT116, HT-29 cell lines	Induction of cellular damage and apoptosis; Epigenetic regulation, alteration of methylation patterns, and transcription; Induction of G2 cell cycle arrest; Upregulation of p62/SQSTM1; Inhibition of the PI3K/Akt pathway.	[22,24,25,26]
Flavokawains	HCT116, HT-29 cell lines	G2/M cell cycle arrest; Induction of autophagy; Induction of apoptosis by increasing intracellular ROS; Activation of p38 MAPK; Upregulation of GADD153, p21 and p27. Downregulation of XIAP, c-IAP1, and c-IAP2; Induction of caspase-dependent and caspase-independent apoptosis pathways; Induction of cytochrome C release; Induction of S and G2/M cell cycle arrest.	[27,32]
Derricin and derricidin	HCT116, DLD-1 cell lines	Inhibition of cell growth; Induction of cell cycle arrest; Regulation of the Wnt/β-catenin signaling pathway.	[33]
Hydroxysafflor Yellow A	HCT116 cell line	Upregulation of E-cadherin; Downregulation of N-cadherin; Downregulation of vimentin; Activation of the PPARγ/PTEN/Akt signaling pathway; Inhibition of PPARγ.	[34]
3-Deoxysappanchalcone	HCT15, HCT116 cell lines	Inhibition of TOPK activity; G2/M cell cycle arrest; Inhibition of cyclin B1; Induction of apoptosis; Activation of p53 and p21.	[36]
Cardamonin	HT-29, HCT116 cell lines	Downregulation of c-Myc and Oct4, Cyclin E, and TSP50; Inhibition of the NF-κB signaling pathway and iNOS signaling; Activation of caspases -3, -9 and Bax; Inhibition of MMP-2, MMP-9, N-cadherin, ADRB2, and EMT; Upregulation of E-cadherin; Reduced colon inflammation.	[39,40,53]
	Metastic animal model ^#^, and CRC mouse model ^§^		
Licochalcone A	HCT116 cell line	Downregulation of PD-L1; Inhibition of the NF-κB and Ras/Raf/MEK pathways; Activation of CTL and T cells; Induction of caspase-dependent apoptosis.	[44]
	Xenograft mouse model *		
Garcinol	HCT116, HT-29 cell lines	Induction of apoptosis; Activation of the Akt and ERK 1/2 pathways; Oxidative stress-mediated pathways; Downregulation of mPGES-1; Decrease of PGE_2_ production; Modulation of the mPGES-1/PGE_2_/HIF-1α pathways.	[46,47]
Isobavachalcone	HCT116, SW480 cell lines	Activation of caspase-3; Cleavage of PARP; Upregulation of Bax; Downregulation of XIAP and survivin; Inhibition of Wnt/β-catenin pathway; Inhibition of Akt and GSK-3β.	[50]
Lonchocarpin	HCT116, SW480, and DLD-1 cell lines	Inhibition of the overactivation of the Wnt/β-catenin pathway Inhibition of viability, cell proliferation, and migration in cancer cell lines; Selective targeting of cancer cells, with no effect on normal intestinal cells.	[52]
	CRC mouse model ^§^		

* The model was established using female athymic nude mice, subcutaneously inoculating CRC cells. ^#^ Metastasis was induced in female BALB/c nude mice via a tail-vein injection of HT-29 cell suspension. ^§^ CRC was induced in mice by administrating azoxymethane (AOM)/dextran sulfate sodium (DSS).

## 3. Challenges Related to Chalcones Administration

**Figure 6:**
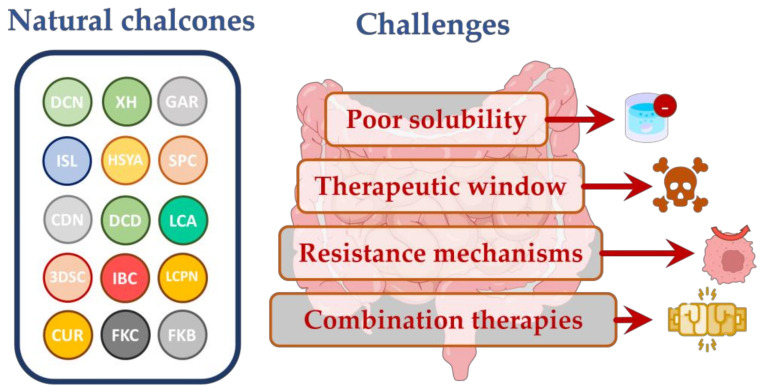
Challenges related to chalcone administration (poor solubility, potential toxicity, cancer cell resistance, and competition issues related to combination therapies).

While chalcones hold immense promise, addressing the associated challenges and limitations is essential to harness their full potential effectively. This section delves into the multifaceted aspects of chalcone research, including its poor solubility, optimal sequencing, resistance mechanisms, and dosage in combination therapies (Figure 6).

### 3.1. Poor Solubility

The poor solubility of chalcones presents a significant challenge when it comes to their administration as potential therapeutic agents [54,55]. One of the primary obstacles posed by poor chalcone solubility is the limited rate and extent of their dissolution in the gastrointestinal tract [56]. This leads to inadequate absorption in the body, resulting in lower plasma concentrations and reduced bioactivity [55]. As a result, higher doses may be required to achieve the desired therapeutic effect, potentially increasing the risks of toxicity and adverse effects. Overcoming the poor solubility of chalcones is essential for harnessing their therapeutic potential and incorporating them into effective pharmaceutical formulations.

### 3.2. Therapeutic Window

The efficacy of chalcones in various therapeutic contexts often hinges on the timing and dosing regimens employed. Achieving the optimal sequencing and dosage is a challenging aspect of chalcone research, as it depends on the specific disease target and the pharmacokinetic properties of the chalcone in question [57].

Determining the therapeutic window for chalcone-based therapies is critical to balance efficacy and safety [57,58]. The therapeutic window represents the range of doses at which a chalcone exerts its desired effects without causing unacceptable toxicity [59]. Finding this balance can be particularly challenging, as different chalcones may have distinct therapeutic windows, as shown in various in vitro and in vivo studies (Table 1).

One approach to optimizing dosing is through dose-escalation studies in clinical trials [58,60]. These studies involve gradually increasing the chalcone dosage in cohorts of patients to identify the maximum tolerated dose and the dose that achieves therapeutic efficacy. However, this process can be time-consuming and resource-intensive.

### 3.3. Resistance Mechanisms

The cancer resistance mechanism involves the overexpression of efflux pumps in cancer cells [48,61]. Efflux pumps can actively remove drugs from the intracellular environment, reducing their intracellular concentrations [62]. Chalcones have garnered significant attention for their ability to sensitize cancer cells to chemotherapy and improve the pharmacokinetics of poorly absorbed cancer drugs. Numerous studies have investigated the potential of chalcones as modulators of resistance to conventional chemotherapy drugs, particularly by targeting multidrug efflux transporters such as P-glycoprotein [63,64,65], multidrug resistance-associated protein 1 [66,67], and breast cancer resistance protein [68,69]. These transporters play a crucial role in drug accumulation within cancer cells and contribute to multidrug resistance (MDR) [61]. Strategies to circumvent efflux pump-mediated resistance include the co-administration of efflux pump inhibitors or the design of chalcone derivatives that provide poor substrates for these pumps.

### 3.4. Combination Therapies

Chalcones are often explored as a possible part of combination therapies with other drugs or treatments [15,16,39,47,70,71]. This strategy aims to enhance their efficacy while minimizing toxicity [15,16,72]. The optimal sequencing and dosage of chalcones in combination therapies depend on multiple factors, including the mechanism of action of the co-administered agents and the potential drug–drug interactions.

Nevertheless, careful consideration is needed when combining chalcones with other drugs, as their pharmacokinetic and pharmacodynamic interactions can be complex [73]. Co-administration may alter chalcone metabolism or create competition for binding sites, affecting their effectiveness [73,74]. Therefore, comprehensive pre-clinical studies and pharmacokinetic modeling are essential to guide the rational design of combination therapies.

## 4. Nanoparticle-Based Delivery Systems for Chalcones

### 4.1. Advantages of Nanoparticles for Chalcone Delivery

This section will explore the remarkable advantages of utilizing nanoparticles (NPs) as a novel delivery system for chalcone compounds. NPs offer myriad benefits that enhance the efficacy, solubility, and potential applications of chalcones in drug delivery, making them an exciting and promising avenue for pharmaceutical research.

#### 4.1.1. Enhanced Drug Stability

One of the most significant advantages of NPs is their ability to enhance drug stability [75]. Conventional drugs often degrade rapidly, making it challenging to maintain their efficacy. NPs can encapsulate drugs, protecting them from environmental factors such as oxidation, light, temperature, moisture, and chemical reactions [76,77]. This preservation of drug integrity extends the shelf life and ensures consistent therapeutic effects.

#### 4.1.2. Prolonged Circulation Time

NPs possess the unique ability to extend the circulation time of drugs within the body [76]. Their small size allows them to evade rapid clearance mechanisms, such as renal filtration, enabling drugs to remain in the bloodstream longer [78]. This prolonged circulation time enhances drug bioavailability and reduces the need for frequent dosing, ultimately improving patient compliance.

#### 4.1.3. Enhanced Cellular Uptake

NPs facilitate the delivery of therapeutic agents to target cells and tissues [79]. Their small size and customizable surface properties enable them to interact favorably with cell membranes, promoting cellular uptake [79,80]. This targeted delivery minimizes off-target effects and enhances the therapeutic efficacy of drugs.

#### 4.1.4. Controlled Release of Therapeutic Agents

Controlling the release of therapeutic agents is crucial to achieving optimal drug efficacy while minimizing side effects. NPs can be engineered to release drugs in a controlled and sustained manner [78,81]. This precise control ensures that therapeutic concentrations are maintained over an extended period, reducing the need for frequent dosing and mitigating adverse reactions [81].

### 4.2. Chalcone-Based NPs for CRC Treatment and Pre-Clinical Studies

One of the primary derivatives of chalcones explored in the context of colon cancer treatment is CUR. Notably, several NP-based formulations, including liposomes, micelles, nanogels, chitosan, and polymeric NPs, have been developed, demonstrating their effectiveness in combatting colon cancer in both in vitro and in vivo studies (Table 2) [82,83,84,85,86,87,88,89,90]. Additionally, innovative delivery systems have emerged. For instance, Ndong Ntoutoume et al. developed CUR-cyclodextrin/cellulose nanocrystal complexes (CUR-CD/CNCx), which have exhibited promising in vitro results, demonstrating lower IC_50_ values and a more significant anti-proliferative effect against HT-29 colon cancer cell lines [91]. Another innovative delivery system that has been studied for its therapeutic efficacy involves PEG-PE micelles co-loaded with CUR and doxorubicin (DOX) and targeted with an anti-GLUT1 antibody against HCT116 human colorectal adenocarcinoma cells, studied both in vitro and in vivo [70]. This approach, when compared to non-targeted counterparts, exhibited a robust killing effect even at low doses of DOX in vitro, while, in established tumors in female nude mice, it led to significant tumor inhibition and improved survival [70]. These innovative NP delivery systems hold great promise in the fight against colon cancer.

The success of CUR nanoformulations in pre-clinical studies has paved the way for clinical trials to assess their real-world potential. These trials are essential to validate the feasibility and effectiveness of CUR nanoformulations in human subjects. Currently, three clinical trials related to CUR nanoformulations are registered on clinicaltrials.gov, as detailed in Table 2.

## 5. Conclusions

Natural chalcones and derivatives are promising candidates for colon cancer treatment. Their potential to modulate crucial signaling pathways in colon cancer development and progression makes them valuable as targeted therapeutics. However, it is essential to acknowledge that while chalcones show promise, more pre-clinical studies are needed to validate their efficacy and safety further. Additionally, the integration of NP-based drug delivery systems presents a novel avenue by which to enhance the effectiveness of chalcones in treating colon cancer. While researchers have made strides in developing encapsulated synthetic chalcones that target various cancer cell lines [92,93,94], their specific applicability in the context of colon cancer and their mechanisms of action remain relatively unexplored. As we move forward, it is imperative for the field to address these gaps through continued research and exploration. A comprehensive understanding of the specific mechanisms underlying chalcones’ effectiveness against colon cancer, along with the development of tailored drug delivery systems, will be pivotal for translating these findings into clinical applications. Future discussions should focus on optimizing the solubility of chalcones, determining the optimal sequencing of therapeutic agents, unraveling the resistance mechanisms, and exploring combination therapies that integrate chalcones and chalcone-based NPs.

In essence, the success of colon cancer treatment is multifaceted, and future research endeavors should strive to unravel the complexities of this promising therapeutic approach. By delving deeper into these aspects, we can pave the way for more targeted, effective, and safe treatments for colon cancer in the years to come.

## Figures and Tables

**Table 2:** Mechanisms of the anti-proliferative action of some chalcone nanoformulations against colon cancer, as seen in pre-clinical studies.

	Nanoformulation Studies			
Chalcone	Model	Nanoformulation	Mechanisms	Reference
Curcumin	HCT116, HCT15, Colo205, DLD-1 cell lines	Lyophilized liposome	Improved solubility and bioavailability; Enhanced cytotoxicity against CRC cell lines (short-term and long-term assays); Preservation of stability through lyophilization; Increased cytotoxic activity against MDR-expressing cell lines.	[83]
	HCT-116 cell line, AOM/DSS animal model	CaCO_3_ encapsulated liposomes (LCC)	pH-sensitive liposomes with CaCO_3_ encapsulation; Enhanced CUR delivery through lysosomal pH sensitivity; Swelling of LCC and rapid CUR release in an acidic medium; Efficient cytosolic accumulation of CUR; Improved antitumor effect in a CRC model (AOM/DSS-induced); Enhanced solubility and cytosolic delivery of CUR with LCC; Ideal carrier for hydrophobic drugs in potential clinical applications.	[84]
	Xenograft mouse model	Chitosan-graft-poly (*N*-vinyl caprolactam) NPs containing gold NPs (Au-CRC-TRC-NPs)	Controlled CUR release and apoptosis induction in cancer cells; Sustained circulation for a week in vivo PK/PD * studies are on Swiss albino mice, with no harm shown to internal organs; Tumor localization and retention for a week in colon tumors; Potential as a multi-responsive nanomedicine for RF-assisted cancer treatment modalities.	[87]
	HCT116, IEC-6, HT-29 cell lines	CaCO_3_ NPs loaded with CUR and protein deacetylase inhibitor QTX125, and coated with hyaluronic acid (CaCO_3_@Cur@QTX125@HA)	Synergistic inhibition of cell growth; Excellent internalization in PDO models **; Increased apoptosis; Decreased tumor marker expression.	[89]
Curcumin and quercetin (4:1)	HCT116, HT-29 cell lines	Shellac nanocapsules	Synergistic antioxidant properties; Synergistic cytotoxicity; Enhanced stability; Enhanced bioavailability.	[90]
	**Pre-Clinical Studies**			
**Trial**	**Nanoformulation**		**Mechanisms**	**NCT Number**
Phase 1	CUR conjugated with plant exosomes		Enhances CUR delivery to colon tissues and tumors.	NCT01294072, 2011
Phase 2	CUR phytosome Meriva^©^		Change in the expression of biomarker β-catenin in adenomatous tissue and normal rectal mucosa. Immunohistochemical expression of NF-κB, Ki-67, and p53.	NCT01948661, 2013
Phase 3	Nanostructured lipid CUR particles		A dietary supplement, in addition to standard chemotherapy, enhances the overall survival, response rate, safety, quality of life, and fatigue scale.	NCT02439385, 2015

* PK/PD model: pharmacokinetic/pharmacodynamic model. ** Patient-derived colorectal carcinoid organ models.

## Data Availability

Not applicable.
